# Movement-related artefacts (MR-ART) dataset of matched motion-corrupted and clean structural MRI brain scans

**DOI:** 10.1038/s41597-022-01694-8

**Published:** 2022-10-17

**Authors:** Ádám Nárai, Petra Hermann, Tibor Auer, Péter Kemenczky, János Szalma, István Homolya, Eszter Somogyi, Pál Vakli, Béla Weiss, Zoltán Vidnyánszky

**Affiliations:** 1 Brain Imaging Centre, Research Centre for Natural Sciences, Budapest, 1117 Hungary; 2 School of Psychology, University of Surrey, Guildford, United Kingdom

**Keywords:** Magnetic resonance imaging, Brain imaging, Imaging techniques

## Abstract

Magnetic Resonance Imaging (MRI) provides a unique opportunity to investigate neural changes in healthy and clinical conditions. Its large inherent susceptibility to motion, however, often confounds the measurement. Approaches assessing, correcting, or preventing motion corruption of MRI measurements are under active development, and such efforts can greatly benefit from carefully controlled datasets. We present a unique dataset of structural brain MRI images collected from 148 healthy adults which includes both motion-free and motion-affected data acquired from the same participants. This matched dataset allows direct evaluation of motion artefacts, their impact on derived data, and testing approaches to correct for them. Our dataset further stands out by containing images with different levels of motion artefacts from the same participants, is enriched with expert scoring characterizing the image quality from a clinical point of view and is also complemented with standard image quality metrics obtained from MRIQC. The goal of the dataset is to raise awareness of the issue and provide a useful resource to assess and improve current motion correction approaches.

## Abstract (summary)

**Table:** 

Measurement(s)	brain measurement
Technology Type(s)	Magnetization-Prepared Rapid Gradient Echo MRI
Factor Type(s)	level of head motion
Sample Characteristic - Organism	Homo sapiens

## Background & Summary

Magnetic resonance imaging (MRI) is a non-invasive imaging method, that has become one of the most dominant techniques to study the structural organization of the human brain^1^. It has also become an integral part of the clinical diagnostic routine, and MRI scans are included in the diagnostic work-up for several neurological and neuropsychiatric conditions due to their high spatial resolution, excellent soft tissue contrast, and lack of ionizing radiation^2–4^.

While patient motion can affect any imaging modality, MRI is especially susceptible to motion artefacts, since it has a relatively long image acquisition time compared to other modalities, such as computed tomography and ultrasonography^5^. Moreover, there is a growing interest in developing approaches sensitive enough to capture subtle neuroradiological changes sometimes even before the onset of the clinical symptoms. These approaches, however, can be also confounded by motion artefacts, which is an issue in highly kinetic participants, especially in the developing^6–8^ and ageing populations^8,9^. Patient motion in MRI is a complex problem, and although faster imaging sequences^10^, special k-space sampling techniques^11,12^, as well as prospective^13^ and post-acquisition retrospective correction methods^14,15^ have been proposed to prevent, reduce, or correct motion artefacts, motion correction is still a developing field with no single method that can be applied effectively in all imaging situations^16^.

MRI artefacts induced by head motion, such as ghosting and blurring^5,17^, are confounding factors with a large impact in neuroscientific research and in clinical practice as well. Head motion has been shown to induce a consistent bias in morphometric estimates of brain structures when using popular brain imaging software packages^7,8,18–20^, mimicking the signs of cortical atrophy^18^. Furthermore, in our recent publication, using images that are also a part of this dataset, we showed that segmentation methods can differ in their reliability when applied to images with motion artefacts^21^. Head motion can also limit the diagnostic utility of brain scans. A study regarding clinical practice has reported the prevalence of repeated sequences due to motion artefacts in clinical MRI examinations to be 19.8%, with an estimated cost of $115,000 per scanner per year^22^.

Retrospective artefact correction methods significantly reduce the need for repeated sequences. These methods are mostly based on deep learning approaches^14,15,23^ that typically require a huge number of image pairs with and without artefacts. In the absence of such datasets, deep learning algorithms are usually trained and validated on simulated data^14,23^ or use special training techniques not requiring paired data^15^. However, to measure the performance of these algorithms on real-world imaging data, a validation set of paired motion-free and motion-corrupted scans from human participants is still critical^24,25^. Such datasets enable the characterization for motion artefact toleration of different software packages and facilitate the development and the validation of artefact correction methods.

In response, Movement-Related ARTefacts (MR-ART) collected T1-weighted 3D structural MRI images of heads of 148 healthy adults covering the adult lifespan. Images have been acquired while staying still and while slight and more excessive head motion (i.e. three sets of data for each participant). We also include the MRIQC^26^ report for each scan to provide general image quality metrics (IQMs), which allows for more granular labelling. It makes our dataset especially valuable that images with different levels of motion have been acquired for the same participants. Finally, we also provide artefact scores obtained from trained neuroradiologists for each scan to characterize the impact of the motion artefacts on the clinical use of the images.

## Methods

### Participants

The brain scans have been acquired between 2019 and 2021, and the data collection included 148 healthy adult volunteers (95 female; age range: 18–75 years; median age: 25.16 years; IQR: 10.50 years) with no reported history of neurological or psychiatric diseases. All participants provided written, informed consent before participation. The research protocol used for collecting the dataset was designed and conducted in accordance with the Hungarian regulations and laws, and was approved by the National Institute of Pharmacy and Nutrition (file number: OGYÉI/70184/2017).

### Image acquisition

**Fig. 1:**
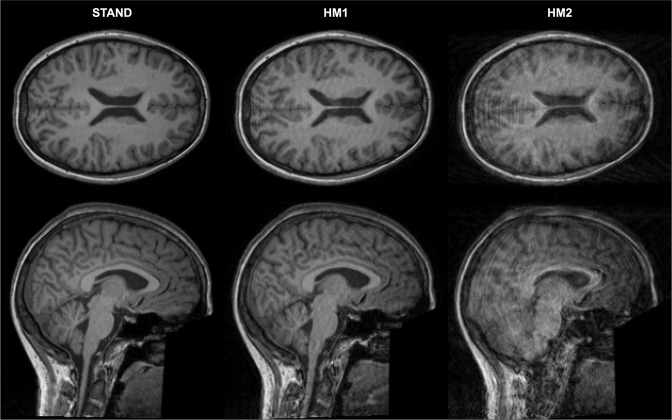
Clean and motion-corrupted images of one representative participant. One axial and one sagittal slice are presented for the standard (STAND) scan, and for scans with low (HM1) and high levels of head motion (HM2). For this participant, the STAND scan was labelled as good (score 1), the HM1 scan as medium (score 2), and the HM2 scan as bad (score 3) quality image from the point of view of clinical diagnostic use.

Image acquisitions were performed on a Siemens Magnetom Prisma 3T MRI scanner (Siemens Healthcare GmbH, Erlangen, Germany) with the standard Siemens 20-channel head-neck receiver coil at the Brain Imaging Centre, Research Centre for Natural Sciences. T1-weighted 3D magnetization-prepared rapid gradient echo (MPRAGE) anatomical images were acquired using 2-fold in-plane GRAPPA acceleration with isotropic 1 mm^3^ spatial resolution (repetition time (TR) = 2300 ms, echo time (TE) = 3 ms, inversion time (TI) = 900 ms, flip angle (FA) = 9°, FOV = 256 × 256 mm). Three T1-weighted structural scans were acquired with the same parameters for each participant in a standard setting without motion (STAND) and with low (HM1) and high levels of head motion (HM2) (see Fig. 1 for images from a representative participant). During the acquisition, a fixation point was presented at the centre of the display, and participants were instructed to gaze at this point. For the STAND scan, participants were instructed not to move at all, while for the HM1 and HM2 scans, participants were instructed to nod their head (tilt it down and then up along the sagittal plane) once every time the word “MOVE” (in Hungarian) appeared on the screen. We used nodding as motion pattern since it is reportedly the most prominent type of head motion, responsible for the majority of motion artefacts^27–31^. To create different levels of motion artefacts, the word “MOVE” was presented for 5 seconds, 5 and 10 times evenly spaced during image acquisition for the HM1 and HM2 scans, respectively. Participants were instructed to avoid lifting their heads from the scanner table while nodding and to return their heads to the original position after performing a nod. Due to acquisition issues, one of the HM1 and HM2 scans is missing for 8 participants.

### Artefact labelling

Labelling was performed based on visual inspection of the structural volumes by two neuroradiologists with more than ten years of experience, who were blind to the acquisition conditions (STAND, HM1 or HM2). Each record was rated on a 3-point scale by one of the neuroradiologists based on image quality from the point of view of clinical diagnostic use. Clinically good (score 1), medium (score 2), and bad quality images (score 3) were differentiated, where bad quality images were considered unusable for clinical diagnostics. The two neuroradiologists initially harmonized their rating on 100 independent structural scans and were encouraged to discuss unclear cases during the whole labelling process in order to make the scores as robust as possible.

## Data Records

The MR-ART dataset is publicly available in the OpenNeuro repository 10.18112/openneuro.ds004173.v1.0.2^32^. The 3D structural images are anonymized and organized according to the Brain Imaging Data Structure (BIDS)^33^. Facial information was removed using PyDeface^34^. STAND (no head motion) scans are marked with the acquisition label “standard”, while HM1 and HM2 (two levels of head motion) scans are marked with the acquisition labels “headmotion1” and “headmotion2”, respectively. Age and sex of each participant are included in the participants.tsv file as per the BIDS standard. The MRIQC results can be found in the /derivatives/mriqc-0.16.1 directory. The IQMs are included in JSON files for each scan, following the BIDS conventions and in a summary file (group_T1w.tsv). MRIQC HTML reports for each scan with filenames identical to the structural scan names and the group level report (group_T1w.html) are also included. Finally, clinical artefact scores are included for each scan in a scans file within the participant folders and in a summary scores file (/derivatives/scores.tsv), with the coding: 1=clinically good, 2=medium, and 3=bad quality images.

## Technical Validation

**Fig. 2:**
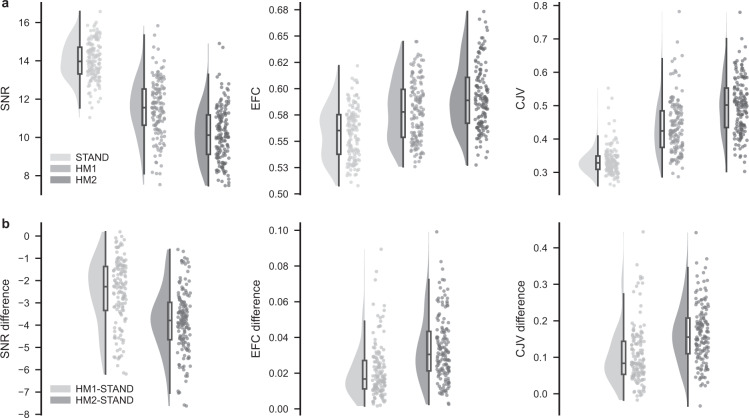
Image quality metrics (IQMs) across acquisition conditions. (**a**) Distribution of three IQMs, namely total signal-to-noise ratio (SNR), entropy focus criterion (EFC), and coefficient of joint variation (CJV) for each acquisition condition (STAND – no head motion, HM1 – low level of head motion, and HM2 – high level of head motion) in our dataset. (**b**) Distribution of within participant differences in IQMs between low head motion and standard (HM1-STAND), as well as high head motion and standard (HM2-STAND) acquisitions.

**Fig. 3:**
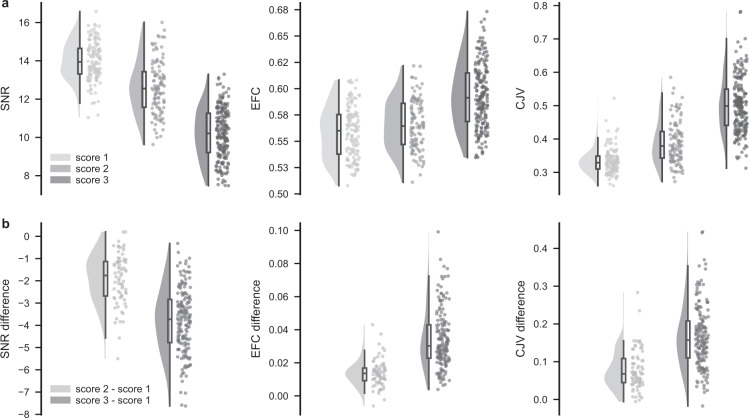
Image quality metrics (IQMs) across clinical artefact scores. (**a**) Distribution of three IQMs, namely total signal-to-noise ratio (SNR), entropy focus criterion (EFC), and coefficient of joint variation (CJV) for each clinical artefact score (score 1 – good, score 2 – medium, and score 3 – bad quality images) in our dataset. (**b**) Distribution of within participant differences in IQMs between medium and good (score 2 - score 1), as well as bad and good (score 3 - score 1) quality images from the point of view of clinical diagnostic use.

**Fig. 4:**
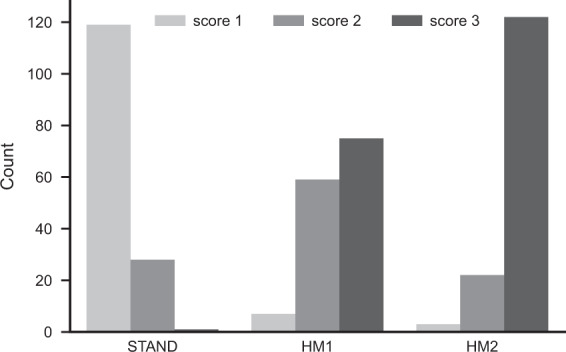
Distribution of clinical artefact scores within each acquisition condition. Barplots show the count of clinical artefact scores (score 1 – good, score 2 – medium, and score 3 – bad quality images) within each acquisition condition (STAND – no head motion, HM1 – low level of head motion, and HM2 – high level of head motion).

Overall image quality and the effect of head motion on image quality were assessed using MRIQC and all the reports are published with the data. MRIQC assesses image quality without reference to any particular standard dataset, which makes the interpretation of the image quality parameters somewhat difficult. The matched design of our dataset, however, allows direct comparison and evaluation of the image quality metrics. Three IQMs, namely total signal-to-noise ratio (SNR), entropy focus criterion (EFC), and coefficient of joint variation (CJV) were chosen to demonstrate and evaluate the image quality differences between acquisition conditions (Fig. 2a) and clinical artefact scores (Fig. 3a). For easier interpretation, we also report within-participant differences in these metrics with reference to the standard acquisition (Fig. 2b) and to the clinically good scan (Fig. 3b). Finally, we included Fig. 4 showing the distribution of clinical artefact scores within each acquisition condition to describe the relationship between the two classifications.

## Usage Notes

The full MR-ART dataset is publicly available in the OpenNeuro repository^32^. We encourage other labs to use this dataset under the requirements of citing this paper and the data citation for the source of the data. The most distinguishing property of this dataset is that each participant has T1-weighted structural MRI scans collected while laying still and with two different levels of the most common form of head movement. This unique property makes the dataset particularly useful for testing the software packages’ sensitivity to motion artefacts and as a real-world test set for motion correction algorithms. While we followed the best common practice to control the amount of motion in each acquisition condition, the measurement conditions correspond to real-world scenarios; therefore, there is some heterogeneity in the dataset, which ensures a good representation of cases, which is crucial for generating generalisable models. We also provide MRIQC IQMs and clinical artefact scores, allowing researchers to control the amount of heterogeneity by using various selection criteria based on the image quality. While the MRIQC IQMs can provide information about generic image quality, clinical scores are further useful for developing and testing automatic brain MRI quality control algorithms for clinical pipelines.

## Data Availability

The described dataset was generated using open-source software packages. The structural images were converted and organized into the BIDS using dcm2bids (v2.1.6)^35^, facial information was removed with PyDeface (v2.0.0)^34^, and IQMs were generated using MRIQC (v0.16.1)^26^. Each software provides extensive user documentation, which were followed to create the dataset.
